# Patient- and proxy-reported outcome measures instruments for the assessment of asthma control among adult and pediatric population: A protocol for systematic review

**DOI:** 10.1097/MD.0000000000020078

**Published:** 2020-05-08

**Authors:** Thayla A. Santino, Karolinne Souza Monteiro, Matheus de Paiva Azevedo, Cecília M. Patino, Sara Ahmed, Karla M.P.P. de Mendonça

**Affiliations:** a Graduate Program of Physical Therapy, Federal University of Rio Grande do Norte, Natal; b Faculty of Health Science of Trairi, Federal University of Rio Grande do Norte, Santa Cruz; c Department of Physical Therapy, Federal University of Rio Grande do Norte, Natal, Brazil; d Department of Preventive Medicine, Keck School of Medicine, University of Southern California, Los Angeles, CA, USA; e Faculty of Medicine, School of Physical and Occupational Therapy, McGill University, Montreal, QC, Canada.

**Keywords:** asthma, measurement properties, patient reported outcomes

## Abstract

**Background:**

Asthma is a chronic airways inflammatory disease considered as a serious public health problem. Since asthma is a lifelong condition, the assessment of its control is important to achieve a better self-management. Based on the advances of the assessment tools, many instruments have been developed to assess asthma control. Therefore, this systematic review aims to assess the measurement properties, the methodological quality, and the content of outcome measures of the available patient- and proxy-reported asthma control instruments.

**Methods:**

This is a systematic review protocol of the measurement properties of asthma control patient- and proxy-reported outcome instruments. Database searches will be primarily performed on MEDLINE, EMBASE, Web of Science, ScienceDirect and PsycINFO. A manual search of websites considered databases for questionnaires and reference lists will also be conducted. The methodological quality of the studies and the measurement properties will be critically appraised using the COSMIN risk of bias (RoB) checklist. The content of all measurement instruments will be compared based on the International Classification of Functioning, Disability and Health framework.

**Results:**

The findings from this systematic review will be disseminated through publication in a peer-reviewed journal and presented at scientific conferences.

**Conclusion:**

The proposed systematic review will produce a comprehensive evaluation of the measurement properties of the currently available asthma control instruments for both adult and pediatric populations. We aim to help researchers and practitioners in their choice of an adequate instrument and to highlight the gaps in currently available tools.

**Systematic review registration:**

PROSPERO registration number: CRD42019126042.

## Abstract 2

Supplemental Digital Content is available in the text

## 1 Introduction

Asthma is a chronic inflammatory disease that has a high prevalence worldwide.^[1]^ This disease tends to be a lifelong condition, which can be developed in early childhood or, less frequently, in adulthood.^[1–3]^ Also known as a serious public health issue, asthma has been associated with increased morbidity and mortality and poses an economic burden as it is associated with high health care costs.^[2,4,5]^ When uncontrolled, asthma can influence physical, mental, emotional and social health, ultimately impacting quality of life, and limiting individuals’ daily activities and school/work participation.^[1,3]^ Beyond personal and health care factors, the condition affects families, caregivers, and society.^[6]^

Despite the availability and advances in asthma treatment and management, most people do not adequately control their symptoms.^[7,8]^ Poor asthma control has been associated with a lack of adherence to medication and therapies failure, presence of comorbidities, psychosocial influences, and misdiagnosis.^[9]^ Therefore, adequate assessment and treatment are the primary components of asthma management.^[10,11]^

Asthma control has been assessed by 2 main domains: symptom control and expected future risk factors.^[1,6]^ Over the last decades, standardized questionnaires were developed and used to evaluate subjective measures of asthma control and management.^[12,13]^ These instruments, mostly patient- or proxy-reported, are commonly used in daily clinical practice and in research to track asthma self-management strategies.^[12]^

There are few published reviews of asthma control questionnaires.^[12,14,15]^ Moreover, some methodological approaches have limited the findings of these previous reviews. Most of the previous reviews do not provide a systematic evaluation of the methodological quality of the studies, the quality of the measurement properties, and information about the content of the items and domains. They also do not include more recently developed instruments.

The proposed systematic review aims to identify, critically appraise, compare, and summarize all available instruments developed to assess asthma control in adult and pediatric populations. Additionally, to the best of our knowledge, this will be the first study to perform a systematic evaluation of the measurement properties, the methodological quality, and the contents of the patient- and proxy-reported asthma control instruments. This systematic review also aims to provide recommendations on the most appropriate questionnaires.

## 2 Methods

### 2.1 Study method

The methods for this systematic review have been developed according to the Preferred Reporting Items for Systematic Reviews and Meta-Analysis Protocol (PRISMA-P) statement^[16]^ and to the COnsensus-based Standards for the selection of health Measurement INstruments (COSMIN).^[17]^ The final review will be reported using the PRISMA statement as a guide.^[18]^

### 2.2 Protocol registration

This systematic review protocol was registered in the International Prospective Register of Systematic Reviews (PROSPERO) (registration number CRD42019126042). Relevant changes to the protocol will be documented in the PROSPERO and published in the final review.

### 2.3 Study inclusion criteria

Studies will be included if: the study uses a patient- or proxy-reported measurement instrument for assessment of asthma control; the study sample represents the population of interest (adult or pediatric population); the study aims to evaluate ≥ measurement properties or the development of a patient- or proxy-reported measurement instrument.

### 2.4 Study exclusion criteria

Studies will be excluded if: patient- or proxy-reported measures were used separately as an outcome measurement instrument, such as clinical trials; or studies in which the patient- or proxy-reported instruments were being used in a validation study of another instrument. Studies will also be excluded if they: are available as abstract only, or are published in any other language than English, Portuguese, Spanish, or French.

### 2.5 Literature search

A systematic electronic search will be performed in the following databases: MEDLINE, EMBASE, Web of Science, ScienceDirect and PsycINFO. A manual search of websites considered databases for questionnaires will also be carried out on: PROQOLID (http://www.proqolid.org), PROMIS (http://www.nihpromis.org), and Medical Outcome Trust (http://www.outcomes-trust.org). The reference list of eligible publications will be manually searched to identify additional relevant studies on this topic.

Database searches will be conducted from the date of inception until the present. The searches will be re-run just before the final analyses to find additional studies that may have been published after the initial search.

### 2.6 Search strategy

Following the COSMIN recommendations, the search strategy will contain blocks of search terms related to these aspects:

1. Construct of interest (asthma control): no search terms for asthma control will be used. Instead, questionnaires measuring asthma control will be selected by hand from the search;
2. Target population (people with asthma): different terms used to identify people with asthma will also be used;
3. Type of instrument (questionnaire): when possible and available, an instrument filter will be used for finding studies on patient- or proxy-reported outcomes or questionnaires;
4. Measurement properties: when possible and available, a validated search filter for studies on outcome measures will be used, which has been developed by Terwee et al.^[19]^

A draft of the search strategy for 1 database is presented in Supplementary Digital Content 1 (see Table, Supplemental Content, which illustrates the search strategy for MEDLINE).

### 2.7 Study selection

Two review authors (TAS, MPA) will independently screen titles and abstracts for inclusion. The full-text of potentially eligible studies will be independently screened. Subsequentially, the review authors will identify studies for inclusion and identify and record reasons for exclusion of the ineligible studies. If required, any disagreement will be resolved through discussion and consultation with a third review author (KMPPM).

The electronic search will be imported into the reference list management tool Mendeley (https://www.mendeley.com). Any duplicates generated by the search strategy will be removed using Mendeley before screening. Then, the reference list will be exported to the Rayyan QCRI systematic review web-based application (https://rayyan.qcri.org).^[20]^ The selection process will be recorded in sufficient detail to complete a PRISMA flow diagram.

### 2.8 Data extraction

A standardized, pre-piloted form will be used to extract data from the included studies. The extracted information will include:

1. General characteristics of the instruments (name, construct, subscales or domains measured, the number of items, source of information [patient- or proxy-reported outcome], method of administration, response options, range of scores, recall period, language versions available, availability);
2. Characteristics of the study population (country, language, age, sex, disease severity, diagnosis criteria, setting, total sample, the method used to select participants, the percentage of response rate, inclusion and exclusion criteria);
3. Results of the measurement properties (eg, reliability, validity, responsiveness to change);
4. Evidence on the interpretability of the included questionnaires and feasibility.

Two review authors (TAS, KSM) will independently extract the data. In case of disagreement, a third review author (KMPPM) will be consulted to reach consensus. Missing data or any other information will be requested from study authors by electronic correspondence.

### 2.9 Assessment of the methodological quality and the measurement properties

The methodological quality of the studies will be critically appraised using the COSMIN RoB Checklist.^[17]^ Two review authors (TAS, MPA) will independently perform a quality assessment of all studies and of all measurement properties evaluated. In cases of disagreement, a third reviewer (KSM) will be consulted. Each study will be evaluated using a 4-point scale (very good, adequate, doubtful or inadequate quality). Subsequently, each questionnaire will be rated by using the updated 3-point scale criteria for good measurement properties (sufficient [+], insufficient [−], and indeterminate [?]).

### 2.10 Assessment of the content

The content of all measurement instruments will be compared based on the International Classification of Functioning, Disability and Health (ICF) framework. Two review authors (TAS, MPA) will independently perform the content evaluation by linking the items of all identified questionnaires to the ICF, in accordance with the ICF linking rules.^[21–23]^ In case of disagreement, we will resolve it by contacting a third reviewer (KSM).

### 2.11 Data synthesis

We will provide a narrative synthesis of the findings from the included studies, structured around the extracted data. We will qualitatively summarize or quantitatively pool the results and findings of different studies that include the measurement properties of the same instrument. The pooling of measurement properties or summarized results will be reported in the Summary of Finding (SoF) tables. Studies will only be pooled if they are sufficiently similar considering the language, source of the information of the instrument (patient- or proxy-reported outcome), study population, and the form of administration. We will evaluate the pooled or summarized results against the criteria of good measurement properties, to determine whether the measurement properties of the questionnaire are sufficient (+), insufficient (−), inconsistent (+/−), indeterminate (?). In addition, to determine the overall quality of evidence, we will apply a modified Grading of Recommendations, Assessment, Development, and Evaluation (GRADE) approach.^[17,24]^ The modified GRADE includes 4 levels of quality of evidence (high, moderate, low, and very low).^[17,24]^

### 2.12 Developing recommendations to select patient- and proxy-reported outcomes instruments

The recommendations will be developed according to COSMIN guideline.^[17]^ If possible, each instrument will be stratified into 3 categories, in which the instrument: will be recommended as the most suitable regarding the construct and population; needs additional validation studies; however, it still has the potential to be recommended; should not be recommended due to a high quality of evidence for an insufficient measurement property.

### 2.13 Ethics and dissemination

This study does not require ethical approval because it will be conducted based on data that have been published. The findings from this systematic review will be disseminated through publication in a peer-reviewed journal and presented at scientific conferences.

## 3 Discussion

The proposed systematic review will present a comprehensive evaluation of the measurement properties of currently available asthma control instruments for both adult and pediatric populations. The findings will highlight gaps and inform the direction of future research in this field. We aim to use a transparent, robust, and systematic process throughout this review. Thus, we will present and discuss the major findings of this systematic review, as well as the strengths and limitations of all reviewed tools. We expect that this systematic review will also help researchers and practitioners in their choice of an adequate instrument to assess asthma control.

## Acknowledgments

The authors thank the Federal University of Rio Grande do Norte, the University of Southern California, and McGill University. The authors also like to thank Coordenação de Aperfeiçoamento de Pessoal de Nível Superior (CAPES) for supporting this study.

## Author contributions

**Conceptualization:** Thayla A Santino, Karolinne Souza Monteiro, Matheus de Paiva Azevedo, Cecília M. Patino, Sara Ahmed, Karla M. P. P. de Mendonça.

**Methodology:** Thayla A Santino, Karla M. P. P. de Mendonça.

**Software:** Thayla A Santino.

**Supervision:** Karla M. P. P. de Mendonça.

**Writing – original draft:** Thayla A Santino, Karolinne Souza Monteiro, Matheus de Paiva Azevedo.

**Writing – review & editing:** Matheus de Paiva Azevedo, Cecília M. Patino, Sara Ahmed, Karla M. P. P. de Mendonça.

## Supplementary Material

Supplemental Digital Content
