# Supplementary material for: Patient- and proxy-reported outcome measures instruments for the assessment of asthma control among adult and pediatric population: A protocol for systematic review
Source: Medicine (Baltimore). 2020 May 8;99(19):e20078. doi: 10.1097/MD.0000000000020078 (PMC7220545; doi:10.1097/MD.0000000000020078)
Supplement: Supplemental Digital Content [file medi-99-e20078-s001.docx]

**Supplemental Digital Content 1.** Table that illustrates a draft of the search strategy for MEDLINE (OVID)

| **#** | **Advanced search** |
| --- | --- |
| 1 | exp Asthma/ |
| 2 | asthma$.tw. |
| 3 | 1 or 2 |
| 4 | “surveys and questionnaires”.mp. |
| 5 | exp “surveys and questionnaires”/ |
| 6 | 4 or 5 |
| 7 | (instrumentation or methods).fs. |
| 8 | (Validation Studies or Comparative Study).pt. |
| 9 | exp Psychometrics/ |
| 10 | psychometr*.ti,ab. |
| 11 | (clinimetr* or clinometr*).tw. |
| 12 | exp “Outcome Assessment (Health Care)”/ |
| 13 | outcome assessment.ti,ab. |
| 14 | outcome measure*.tw. |
| 15 | exp Observer Variation/ |
| 16 | observer variation.ti,ab. |
| 17 | exp Health Status Indicators/ |
| 18 | exp “Reproducibility of Results”/ |
| 19 | reproducib*.ti,ab. |
| 20 | exp Discriminant Analysis/ |
| 21 | (reliab* or unreliab* or valid* or coefficient or homogeneity or homogeneous or “internal consistency”).ti,ab. |
| 22 | (cronbach* and (alpha or alphas)).ti,ab. |
| 23 | (item and (correlation* or selection* or reduction*)).ti,ab. |
| 24 | (agreement or precision or imprecision or “precise values” or test-retest).ti,ab. |
| 25 | (test and retest).ti,ab. |
| 26 | (reliab* and (test or retest)).ti,ab. |
| 27 | (stability or interrater or inter-rater or intrarater or intra-rater or intertester or inter-tester or intratester or intra-tester or interobserver or inter-observer or intraobserver or intraobserver or intertechnician or inter-technician or intratechnician or intra-technician or interexaminer or inter-examiner or intraexaminer or intra-examiner or interassay or interassay or intraassay or intra-assay or interindividual or inter-individual or intraindividual or intra-individual or interparticipant or inter-participant or intraparticipant or intra-participant or kappa or kappa’s or kappas or repeatab*).ti,ab. |
| 28 | ((replicab* or repeated) and (measure or measures or findings or result or results or test or tests)).ti,ab. |
| 29 | (generaliza* or generalisa* or concordance).ti,ab. |
| 30 | (intraclass and correlation*).ti,ab |
| 31 | (discriminative or “known group” or factor analysis or factor analyses or dimension* or subscale*).ti,ab. |
| 32 | (multitrait and scaling and (analysis or analyses)).ti,ab |
| 33 | (item discriminant or interscale correlation* or error or errors or “individual variability”).ti,ab. |
| 34 | (variability and (analysis or values)).ti,ab |
| 35 | (uncertainty and (measurement or measuring)).ti,ab. |
| 36 | (“standard error of measurement” or sensitiv* or responsive*).ti,ab. |
| 37 | ((minimal or minimally or clinical or clinically) and (important or significant or detectable) and (change or difference)).ti,ab. |
| 38 | (small* and (real or detectable) and (change or difference)).ti,ab. |
| 39 | (meaningful change or “ceiling effect” or “floor effect” or “Item response model” or IRT or Rasch or “Differential item functioning” or DIF or “computer adaptive testing” or “item bank” or “cross-cultural equivalence”).ti,ab. |
| 40 | 7 or 8 or 9 or 10 or 11 or 12 or 13 or 14 or 15 or 16 or 17 or 18 or 19 or 20 or 21 or 22 or 23 or 24 or 25 or 26 or 27 or 28 or 29 or 30 or 31 or 32 or 33 or 34 or 35 or 36 or 37 or 38 or 39 |
| 41 | (child* or pediatric* or infan* or adolescent* or adult*).mp. |
| 42 | 3 and 6 and 40 and 41 |
